# Impact of obstructive sleep apnea complicated with type 2 diabetes on long-term cardiovascular risks and all-cause mortality in elderly patients

**DOI:** 10.1186/s12877-021-02461-x

**Published:** 2021-09-25

**Authors:** Xiaofeng Su, Jian Hua Li, Yinghui Gao, Kaibing Chen, Yan Gao, Jing Jing Guo, Min Shi, Xiao Zou, Weihao Xu, Li Bo Zhao, Huanhuan Wang, Yabin Wang, Juan Liu, Hu Xu, Xiaoxuan Kong, Junling Lin, Xiaoshun Qian, Jiming Han, Lin Liu

**Affiliations:** 1 Medical College, Yan’an University, Yan’an, Shanxi Province China; 2 Cardiology Department of the Second Medical Center & National Clinical Research Center for Geriatric Diseases, Chinese PLA General Hospital, Beijing, China; 3 PKU-UPenn Sleep Center, Peking University International Hospital, Beijing, China; 4 Sleep Center, The Affiliated Hospital of Gansu University of Chinese Medicine, Lanzhou City, Gansu Province China; 5 Department of General Practice, 960th Hospital of PLA, Jinan, Shandong Province China; 6 Sleep Medicine Center, Department of Respiratory and Critical Care Medicine, Peking University People’s Hospital, Beijing, China; 7 Department of Respiratory and Critical Care Medicine of the Second Medical Center & National Clinical Research Center for Geriatric Diseases, Chinese PLA General Hospital, 28 Fuxing Road, Beijing, China; 8 Department of Respiratory and Critical Care Medicine, Beijing Chaoyang Hospital Affiliated to Capital Medical University, Beijing, China

**Keywords:** Obstructive sleep apnea, Type 2 diabetes, Elderly, Major adverse cardiovascular events, Mortality, Cardiovascular disease

## Abstract

**Background:**

The prognostic significance of obstructive sleep apnea (OSA) in elderly patients with type 2 diabetes is unclear. The aim of this study was to determine the risk of cardiovascular disease (CVD) and mortality in elderly patients with OSA complicated with type 2 diabetes compared to patients with OSA without type 2 diabetes.

**Methods:**

From January 2015 to October 2017, 1113 eligible elderly patients with OSA, no history of cardiovascular, ≥60 years of age, and complete follow-up records were enrolled in this consecutive multicentre prospective cohort study. All patients had completed polysomnography (PSG) examinations. An apnoea-hypopnoea index of ≥5 events per hour recorded by polysomnography was defined as the diagnostic criterion for OSA. We collected baseline demographics, clinical characteristics, sleep parameters and follow-up outcomes. The primary aim of this study was to identify the risk of incident major adverse cardiovascular events (MACE). Secondary outcomes were all-cause mortality, components of MACE and a composite of all events. Kaplan-Meier survival analysis and Cox proportional hazards models were used to evaluate whether type 2 diabetes was associated with incident events.

**Results:**

A total of 266 (23.9%) patients had OSA complicated with type 2 diabetes. MACE occurred in 97 patients during the median 42-month follow-up. Kaplan-Meier survival curves indicated a significant relationship between type 2 diabetes and MACE (log-rank *P* = 0.003). Multivariable Cox regression analysis showed that type 2 diabetes increased the risk of MACE (HR = 1.64, 95% CI:1.08–2.47, *P* = 0.019), hospitalisation for unstable angina (HR = 2.11, 95% CI:1.23–3.64, *P* = 0.007) and a composite of all events in elderly patients with OSA (HR = 1.70, 95% CI:1.17–2.49, *P* = 0.007). However, there were no significant differences in the incidence of cardiovascular death, all-cause mortality, MI and hospitalisation for heart failure between patients with and without diabetes (*P* > 0.05). The subgroup analysis demonstrated that females (AHR = 2.46, 95% CI:1.17–5.19, *P* = 0.018), ≥ 70 years (AHR = 1.95, 95% CI:1.08–3.52, *P* = 0.027), overweight and obese (AHR = 2.04, 95% CI:1.29–3.33, *P* = 0.002) with mild OSA (AHR = 2.42, 95% CI: 1.03–5.71, *P* = 0.044) were at a higher risk for MACE by diabetes.

**Conclusion:**

OSA and type 2 diabetes are interrelated and synergistic with MACE, hospitalisation for unstable angina and a composite of all events development. Overweight and obese females, ≥ 70 years with mild OSA combined with type 2 diabetes presented a significantly high MACE risk.

**Supplementary Information:**

The online version contains supplementary material available at 10.1186/s12877-021-02461-x.

## Background

OSA is a chronic and fatal sleep disorder, and OSA-related CVD and mortality worsen the quality of life in patients with OSA [1]. Some studies show that OSA is associated with an increased risk of death and cardiovascular disease [2, 3]. Type 2 diabetes is a frequent comorbidity in patients with OSA [4–6]. Intermittent hypoxaemia and sleep fragmentation in OSA could contribute independently to the development of insulin resistance, glucose intolerance and type 2 diabetes. Conversely, type 2 diabetes may increase predisposition to, or accelerate the progression of OSA, possibly through the development of peripheral neuropathy and abnormalities of ventilatory and upper airway neural control [7, 8]. The prevalence of OSA in patients with type 2 diabetes ranges from 50 to 80% [9, 10]. A cross-sectional study confirmed an association between OSA and type 2 diabetes [11]. A longitudinal study showed that OSA patients with type 2 diabetes have higher CVD mortality [12], while another study revealed that people with type 2 diabetes do not seem to have an increased risk of death and myocardial infarction than the general population [13]. Some researchers found that respiratory sleep disorders in the young, middle-aged patients were independent of atrial fibrillation, but there was no association in elderly patients [14]. From a physiological perspective, the elderly have greater hypoxic tolerance, and repeated intermittent hypoxia protects the myocardium against ischaemic injury [15]. A previous study showed that the cardiovascular disease mortality rate for OSA patients younger than 50 years was higher, but the risk significantly reduced after 50 years [16]. Other researchers confirmed that OSA in elderly patients was not related to the increased risk of cardiovascular disease [17]. Thus, the posed question was whether type 2 diabetes complications increase the risk of CVD, all-cause mortality and a composite of all events in patients with OSA, especially in the elderly OSA population.

Therefore, we investigated the association between type 2 diabetes and the incidence of MACE, all-cause mortality and a composite of all events in patients with OSA using the multicentre population-based prospective cohort data.

## Methods

### Study design and participants

The study is a large-scale, multi-center, prospective, cohort study to assess the association of type 2 diabetes with cardiovascular risks and all-cause mortality in elderly patients with OSA. We Consecutive enrolled 1290 elderly patients with OSA from January 2015 to October 2017. All patients were hospitalized at the sleep medicine center of six hospitals, respectively, which including Chinese PLA General Hospital, Peking University International Hospital, Peking University People’s Hospital, Beijing Chaoyang Hospital, 960th Hospital of PLA, and the affiliated Hospital of Gansu University of Chinese Medicine. Patients with OSA, no history of myocardial infarction (MI) or hospitalisation for unstable angina or heart failure, aged ≥60 years, and had completed polysomnography (PSG) examinations were eligible for inclusion. OSA was defined as an apnea-hypopnea index of ≥5 events per hr. The AHI was defined as the number of apnoea and hypopnea per hour of sleep. We excluded 177 patients based on the following criteria: (1) diagnosis of type 1 diabetes between 2015 and 2017 (*n* = 48); (2) one standard treatment of continuous positive airway pressure (CPAP) for OSA (*n* = 71); (3) previous history of myocardial infarction (MI), hospitalisation for unstable angina or heart failure (*n* = 34); (4) presence of malignant tumours (*n* = 3); (5) presence of mental disorders (*n* = 4). Furthermore, we excluded those lost during the follow-up (*n* = 17); the final study subjects were 1113 aging patients with OSA. This study conformed to the STROBE (Strengthening the Reporting of Observational studies in Epidemiology) guidelines and was carried out in accordance with the Declaration of Helsinki. The Ethics Committee of PLA General Hospital (S2019–352-01) approved the study. Written informed consent was available from all participants.

### Polysomnography (PSG)

All patients underwent an overnight sleep monitoring (from 21: 00 to 7: 00 the next day) after clinical stabilization during hospitalization at sleep centre (within 1 weeks after admission) and sleep parameters were recorded using portable laboratory-based polysomnography (PSG) instrument (Compumedics, Melbourne, Australia), as described previously [18]. Sleep parameters from PSG were as follows: continuous polygraphic recording from surface leads for electroencephalography, electrooculography, electromyography, electrocardiography, thermistors for nasal and oral airflow, thoracic and abdominal impedance belts for respiratory effort, pulse oximetry for oxyhaemoglobin concentration, tracheal microphone for snoring and a sensor for the position during sleep. Analysis of sleep tests was scored according to the American Academy of Sleep Medicine 2017 guidelines [19]. Patients PSG records of each hospital were automatically analysed and manually calibrated by manually twice (by two professional sleep technologist), both of whom were blinded to the demographic and clinical characteristics. Further analysis was performed in cases of discrepancy by a senior physician in sleep medicine. OSA was defined as AHI ≥5 events/hour. Patients with AHI < 5 events/hour were considered as the non- OSA. Patients with OSA (AHI ≥5), particularly those with excessive daytime sleepiness, were referred to the sleep center for further evaluation. An apnea was defined as the continuous cessation of airflow for more than 10 s, whereas a hypopnea was defined as a reduction in airflow by 50% with a duration of at least 10 s or a reduction of airflow or respiratory effort by 30% for more than 10 s, accompanied by an electroencephalographic arousal, a 4% or greater oxygen desaturation, or both. The AHI was defined as the number of apnoea and hypopnoea per hour of sleep. The oxygen desaturation index (ODI) was defined as a SaO2 drop of ≥3%. OSA was classified as mild (AHI of 5 to 14.9), moderate (AHI of 15 to 30) or severe (AHI>30) [19, 20].

### Covariates

The following potential confounders and risk factors were extracted from clinical data: age, sex, body mass index (BMI), blood pressure (BP), plasma glucose, HbA1c and self-reported smoking and alcohol use. Sleep parameters were as follows: AHI, ODI, mean oxygen saturation and lowest oxygen saturation. Comorbidities were identified at baseline (carotid atherosclerosis, hyperlipidaemia, atrial fibrillation, hypertension [HTN], chronic obstructive pulmonary disease [COPD], coronary heart disease [CHD] and diabetes from the hospital administrative database over a six-month period before the diagnostic sleep study. These data were assessed by an interviewer who administered the Unified epidemiological questionnaire and were reviewed by three physicians. Blood was drawn for biochemical analysis after overnight fasting. Plasma glucose, 2 h glucose concentration in the standard 75 g oral glucose tolerance test were measured using a Roche C8000 Automatic Analyzer. The categories of covariates were listed in Supplementary Table s-1.

### Definitions

Body mass index (BMI) was calculated as weight (kg) divided by height (m^2^). Current smoking was defined as at least one cigarette a day, and current drinking was defined as drinking once per week for at least half a year. Systolic blood pressure (SBP) and diastolic blood pressure (DBP) were measured three times. Hypertension was defined present if the mean of at least two consecutive measurements of SBP/DBP ≥140/90 mmHg or the use of antihypertension medication [21]. Dyslipidemia using Chinese Guideline for the management of hyperlipidemia in adults was defined as (1) serum cholesterol levels ≥4.7 mmol/L, (2) triglyceride levels ≥2.3 mmol/L, or (3) low-density lipoprotein levels ≥4.1 mmol/L. Patients who meet one of these three items are defined as having hyperlipidemia [22]. Atrial fibrillation was defined based on the ESC 2016 guidelines [23]. Carotid atherosclerosis, coronary heart disease (CHD) and chronic obstructive pulmonary disease (COPD) were determined by a record of a relevant diagnostic clinical (Read) code indicating the presence of the condition [24].

### Diagnostic criteria

We considered any of the following parameters for a diagnosis of type 2 diabetes: (1) diabetes symptoms (typical symptoms, including polydipsia, polyuria and unexplained weight loss) and plasma glucose ≥11.1 mmol/L (200 g/L) at any time; (2) fasting plasma glucose ≥7.0 mmol/L (126 g/L); (3) OGTT2h plasma glucose ≥11.1 mmol/L (200 g/L) [25].

### Treatment and management

The subjects with OSA were split into diabetes group (*n* = 266) and non-diabetes group (*n* = 847). All patients in diabetes group treated by taking diabetes medications. And 1113 patients received standard care during OSA hospitalization at the sleep medicine center of six hospitals according to current guidelines [19]. Patients with AHI < 5 events/hour were seen as the non- OSA. Patients with OSA (AHI ≥5), particularly those with excessive daytime sleepiness, were referred to the sleep center for further evaluation.

### Follow-up

One thousand one hundred thirteen patients with OSA were followed up from the diagnosed time of PSG assessment to December 2020 for MACE, cardiovascular death, all-cause mortality, MI, hospitalisation for unstable angina or heart failure and the development of composite of all events and was performed at 1 month, 3 months, 6 months, 1 year, and then every 6 months thereafter (at least 3 months and up to 1 years). The participants’ outcomes were collected by a clinic visit, medical chart review, or telephone calls by two investigators who were blinded to patients’ PSG results every 6 months. All clinical events were confirmed by source documentation and were adjudicated by the clinical event committee. For the current study, all patients received standard health care depending on their disease status during a median follow-up of 42 months. The primary outcome was MACE, including cardiovascular death, MI and hospitalisation for unstable angina or heart failure. Secondary outcomes were all-cause mortality, individual components of MACE and a composite of all events. The study ended if patients followed up the incidence of new-onset major adverse cardiovascular events (MACE) or all-cause mortality, which was the first MACE or all-cause mortality event for that patient. Two or more MACE or all-cause mortal event were uniformly counted as one event, with the first event time and event reported as the outcome.

### Statistical analysis

Demographics, clinical characteristics and sleep parameters in the study subjects were categorised according to type 2 diabetes using a Pearson’s Chi-square test and an independent t-test. Data were indicated as percentages for categorical variables or mean ± standard deviation for normally distributed continuous variables. Skewed variables were presented as median (interquartile range) and compared using the Mann–Whitney U test. Crude and adjusted hazard ratios (AHR) and their corresponding 95% confidence intervals (CI) for the association between type 2 diabetes and incidence of all events were calculated using Cox proportional hazards regression models. Kaplan-Meier curves were used to visualise the association between type 2 diabetes and adverse events. Two Cox proportional hazards regression models were conducted to examine the association between type 2 diabetes coexisting with OSA and long-term cardiovascular risks and all-cause mortality. Model 1, unadjusted analysis; Model 2(adjusted analysis), Model 1 plus sex, BMI, plasma glucose, alcohol use, HbA1c, WHR, waist circumference, comorbidities of CHD, hyperlipidemia, hypertension, carotid atherosclerosis, atrial fibrillation, and sleep parameters of ODI, TST, T90, TSA90, WHR, waist circumference, AHI, average apnea time and maximum apnea time. All analyses were conducted using the SPSS statistical software (version 25.0, SPSS Inc., Chicago, Illinois, USA).

## Results

### Baseline characteristics

**Fig. 1:**
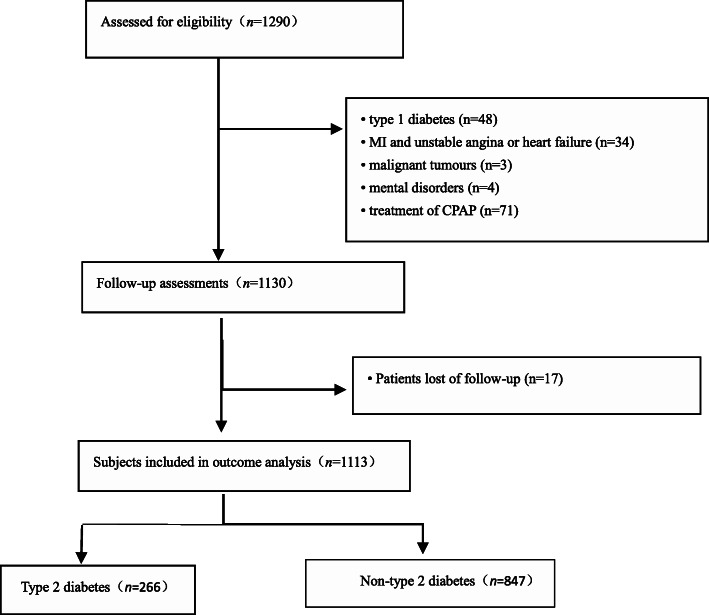
Study flowchart. OSA, obstructive sleep apnea; CPAP indicates continuous positive airway pressure

In total, 1290 consecutive eligible elderly patients with OSA were prospectively enrolled, all of whom underwent a successful overnight sleep study. After exclusion of patients according to predefined criteria, 1113 study subjects with OSA aged ≥60 years in the final analysis (Fig. 1); 285 patients (25.6%) had mild OSA, 336 (30.2%) had moderate OSA, 492 (44.2%) had severe OSA and 266 (23.9%) had OSA complicated with type 2 diabetes. Diabetes patients had higher severe OSA rates than non-diabetes patients (*P* = 0.011). Table 1 present the general characteristics of study participants according to type 2 diabetes. The proportions of alcohol use (13.2% vs. 7.5%), comorbidities (CHD) [39.8% vs. 16.5%], hyperlipidaemia [47.7% vs. 21.8%], hypertension [81.6% vs. 57.7%], atrial fibrillation [11.6% vs. 4.5%] and carotid atherosclerosis [37.2% vs. 22.1%]) in individuals with diabetes were significantly higher than in those without diabetes in patients with OSA. Patients with diabetes also showed significantly higher levels of average systolic (140 mmHg vs. 130 mmHg) and diastolic (80 mmHg vs. 76 mmHg) BP, plasma glucose (6.47 mmol/L vs. 5.18 mmol/L), age (67 year vs. 65 year), waist circumference (91 mm vs. 89 mm), WHR (0.92 vs. 0.87), average apnea time (22.72 s vs. 21.91 s), AHI (30.3 times/h vs. 25.4 times/h), BMI (27.27 kg/m^2^ vs. 25.95 kg/m^2^) and HbA1c (38.44 mmol/mol vs. 36.80 mmol/mol).

**Table 1:** General characteristics of study subjects according to type 2 diabetes

	Total (*n* = 1113)	Diabetes (*n* = 266)	Non-diabetes (*n* = 847)	*P*-Value
Demographics				
Age, y	66.0 (62.0, 71.0)	67 (64.0, 72.0)	65.0 (62.0, 70.0)	0.000
Male, n (%)	675 (60.6)	171 (64.3)	504 (59.5)	0.164
BMI, kg/m^2^	26.30 (23.88, 28.80)	27.27 (24.50, 29.80)	25.95 (23.63, 28.31)	0.000
Waist circumference, cm	90 (80, 99)	91 (80, 101)	89 (80, 99)	0.010
Neck circumference, cm	39 (35, 41)	38 (36, 40)	38 (35,40)	0.653
WHR	0.88 (0.78, 1.01)	0.92 (0.79, 1.05)	0.87 (0.78, 0.98)	0.019
SBP, mmHg	130 (122, 143)	140 (130, 160)	130 (120, 140)	0.000
DBP, mmHg	76 (70, 83)	80 (70, 87)	76 (70, 82)	0.003
Smoking, n (%)	160 (14.4)	31 (11.7)	129 (15.2)	0.252
Drinking, n (%)	98 (8.8)	35 (13.2)	63 (7.5)	0.010
Plasma glucose, mmol/L	6.18 (5.38,6.19)	6.47 (6.04,7.91)	5.18 (4.57,5.90)	0.048
HbA1c, %	5.52 (5.10,5.63)	5.67 (5.44,6.43)	4.32 (4.04,5.62)	0.045
HbA1c, mmol/mol	36.81 (32.23,36,9)	38.44 (36.10,46.70)	36.80 (31.61,37.83)	0.043
Sleep parameters				
AHI, events/h	26.7 (14.6, 45.2)	30.3 (17.2, 48.7)	25.4 (14.0, 44.3)	0.010
ODI, events/h	21.4 (10.2, 40.5)	22.9 (10.9, 41.5)	20.7 (10.1, 39.8)	0.467
MSpO_2_, %	93 (92, 95)	94 (92, 95)	93 (92, 95)	0.184
LSpO_2_, %	80 (72, 85)	80 (70, 86)	81 (73, 85)	0.367
TST, h	7.03 (6.11, 7.42)	7.09 (6.16, 7.43)	7.01 (5.96, 7,42)	0.139
TSA90, min	14.11 (2.28, 60.32)	14.19 (2.48, 60.93)	12.73 (1.98, 56.81)	0.179
T90, %	3.51 (0.61, 15.37)	3.51 (0.64, 15.81)	3.29 (0.49, 14.49)	0.272
Maximum apnea time, s	63.12 (42.44, 85.20)	63.51 (41.96, 86.13)	61.80 (42.11, 84.23)	0.722
Average apnea time, s	22.47 (19.46, 25.46)	22.72 (19.46, 25.68)	21.91 (19.46, 25.10)	0.095
Medical history, n (%)				
Severity of OSA				0.030
Mild OSA	285 (25.6)	55 (20.7)	230 (27.2)	
Moderate OSA	336 (30.2)	76 (28.6)	260 (30.7)	
Severe OSA	492 (44.2)	135 (50.8)	357 (42.1)	
CHD	246 (22.1)	106 (39.8)	140 (16.5)	0.000
Hyperlipidemia	312 (28.0)	128 (47.7)	185 (21.8)	0.000
Hypertension	706 (63.4)	217 (81.6)	489 (57.7)	0.000
Atrial fibrillation	69 (6.2)	31 (11.6)	38 (4.5)	0.000
Carotid atherosclerosis	286 (25.7)	99 (37.2)	187 (22.1)	0.000
COPD	78 (7.0)	22 (8.3)	56 (6.6)	0.355

*BMI* body mass index, *WHR* waist/hip ratio, *SBP* systolic blood pressure, *DBP* diastolic blood pressure, *AHI* the apnea-hypopnea index, *ODI* the oxygen desaturation index, *MSpO2* the mean pulse oxygen saturation, *LSpO2* the lowest pulse oxygen saturation, *TSA90* the duration of time with SaO2 < 90%, *T90* percentage of the times for SaO_2_<90*%* in total monitoring time during overnight sleep, *OSA* obstructive sleep apnea, *CHD* coronary heart disease, *COPD* chronic obstructive pulmonary disease

### Impact of type 2 diabetes on adverse events during follow up

#### Primary outcome: MACE

**Fig. 2:**
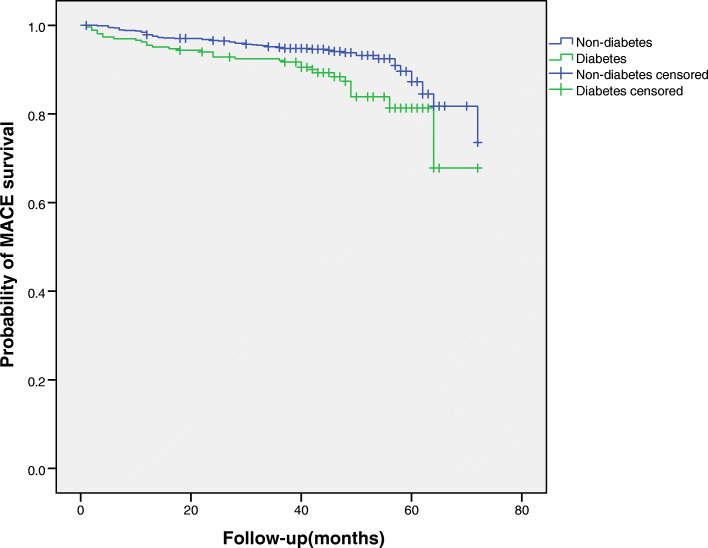
Kaplan-Meier estimates of probability of survival (%) for MACE (Primary end point). Log-rank test: *P* = 0.003. MACE: major adverse cardiovascular event

Crude numbers of adverse events are shown in Table 2. This study examined 97 events of MACE (8.7%) during a median follow-up period of 42 months (range 1 to 72 months): 33 (12.4%) in diabetes patients and 64 (7.6%) in non-diabetes patients. Kaplan-Meier analysis showed that the cumulative event rate of MACE among OSA patients with type 2 diabetes was significantly higher than in OSA patients without type 2 diabetes (Log-rank test: *P* = 0.003) (Fig. 2). Table 3 showed unadjusted and adjusted HRs for incidence of MACE according to diabetes with OSA. Following adjustment for sex, BMI, plasma glucose, alcohol use, HbA1c, ODI, TST, T90, TSA90, WHR, waist circumference, AHI, average apnea time, maximum apnea time, and comorbidities of CHD, hyperlipidaemia, hypertension, carotid atherosclerosis, atrial fibrillation and diabetes significantly increased the risk of MACE (HR = 1.64, 95% CI:1.08–2.47, *P* = 0.019) in elderly patients with OSA. In the subgroup analysis, adjusted hazard ratios for MACE by diabetes were higher in overweight and obese females ≥70 years and patients with mild OSA (Table 4).

**Table 2:** Crude number of adverse events during follow-up

Follow-up outcomes	Total (*n* = 1113)	Diabetes (*n* = 266)	Non- diabetes (*n* = 847)
MACE, n (%)	97 (8.7)	33 (12.4)	64 (7.6)
Cardiovascular death, n (%)	20 (1.8)	6 (2.3)	14 (1.7)
MI, n (%)	26 (2.3)	10 (3.8)	16 (1.9)
Hospitalization for unstable angina, n (%)	56 (5.0)	23 (8.6)	33 (3.9)
Hospitalization for heart failure, n (%)	10 (0.9)	3 (1.1)	7 (0.8)
All-cause mortality, n (%)	43 (3.9)	15 (5.6)	28 (3.3)
Composite of all events, n (%)	119 (10.7)	41 (15.5)	78 (9.2)

*MACE* major adverse cardiovascular event, *MI* myocardial infarction

**Table 3:** Association between type 2 diabetes and incidence of all events

	Unadjusted analysis		Adjusted analysis	
	*HR* (95*% CI*)	*P*-Value	*HR* (95*% CI*)	*P*-Value
MACE	1.89 (1.24–2.88)	0.003	1.64 (1.08–2.47)	0.019
Cardiovascular death	1.58 (0.60–4.13)	0.353	1.27 (0.48–3.36)	0.629
MI	2.59 (1.17–5.75)	0.019	2.01 (0.87–4.64)	0.103
Hospitalization for unstable angina	2.42 (1.42–4.13)	0.001	2.11 (1.23–3.64)	0.007
Hospitalization for heart failure	1.55 (0.40–6.04)	0.529	1.30 (0.33–5.08)	0.713
All-cause mortality	2.02 (1.07–3.80)	0.029	1.54 (0.82–2.90)	0.187
Composite of all events	2.01 (1.38–2.95)	0.000	1.70 (1.17–2.49)	0.007

*MACE* major adverse cardiovascular event, *MI* myocardial infarction

**Table 4:** Subgroup analysis of the associations between type 2 diabetes and MACE

	Unadjusted analysis		Adjusted analysis	
	*HR* (95*%CI*)	*P*-Value	*HR* (95*%CI*)	*P*-Value
Age				
<70	1.62 (0.86–3.07)	1.623	1.41 (0.73–2.69)	0.307
≥ 70	1.86 (1.04–3.32)	0.036	1.95 (1.08–3.52)	0.027
Severity of OSA				
Mild	2.48 (1.10–5.61)	0.029	2.42 (1.03–5.71)	0.044
Moderate-severe	1.76 (1.07–2.89)	0.025	1.68 (0.77–3.65)	0.192
Gender				
Male	1.71 (1.01–2.90)	0.045	1.66 (0.98–2.81)	0.62
Female	2.31 (1.13–4.70)	0.022	2.46 (1.17–5.19)	0.018
BMI				
Normal (18.5–22.9)	1.60 (0.46–5.56)	0.463	1.90 (0.53–6.86)	0.326
Overweight and obese (≥23)	2.02 (1.28–3.18)	0.003	2.04 (1.29–3.33)	0.002

*BMI* body mass index, *OSA* obstructive sleep apnea

#### Secondary outcomes: all-cause mortality, components of MACE, and a composite of all events

**Fig. 3:**
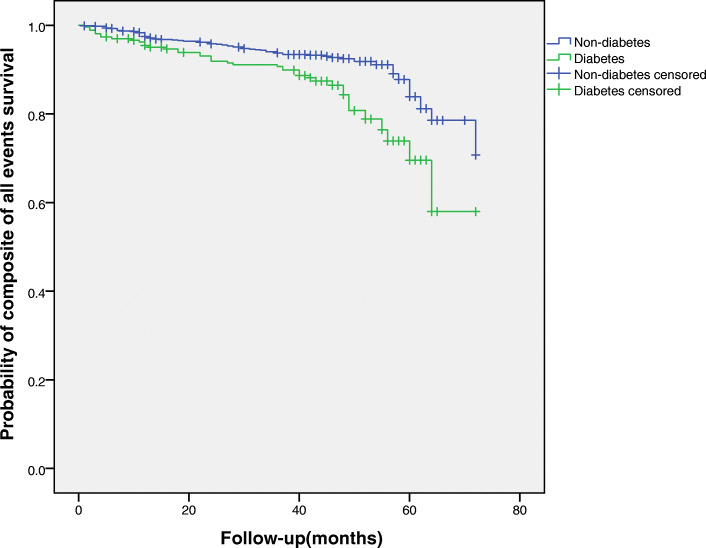
Kaplan-Meier estimates of probability of survival (%) for composite of all events. Log-rank test: *P* = 0.000

**Fig. 4:**
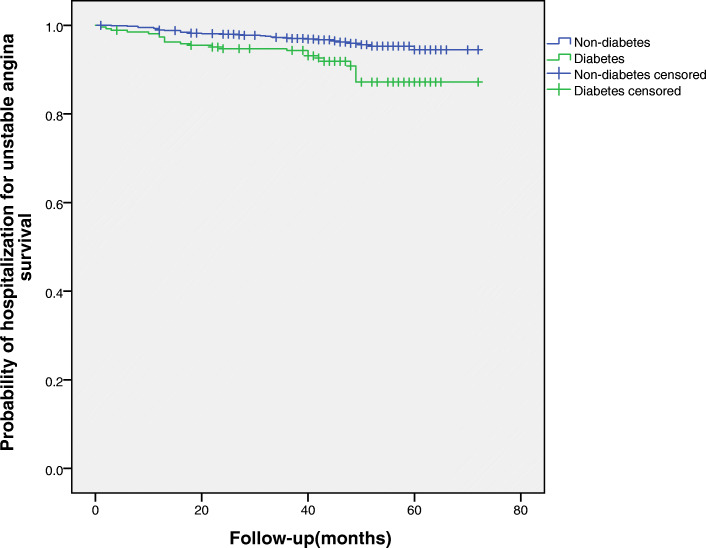
Kaplan-Meier estimates of probability of survival (%) for Hospitalization for unstable angina. Log-rank test: *P* = 0.026

Forty-three patients died during the follow-up period, the proportions of diabetes group vs. non-diabetes group (5.6% vs. 3.3%), Table 2. The univariate analysis showed that diabetes was associated with a higher (approximately 4-year) risk of all-cause mortality in elderly patients with OSA (HR = 2.02, 95% CI: 1.07–3.80, *P* = 0.029). However, for adjusted hazard ratios for all-cause mortality, the trend of increased risk was statistically insignificant (HR = 1.54, 95% CI: 0.82–2.90, *P* = 0.187), Table 3. In the adjusted Cox regression analysis, there were no significant differences in the incidence of cardiovascular death, MI and hospitalisation for heart failure between patients with and without diabetes (*P* > 0.05), Table 3. However, multivariable Cox regression analyses showed that diabetes significantly increased the risk of a composite of all events (HR = 1.70, 95% CI: 1.17–2.49, *P* = 0.007) and hospitalisation for unstable angina (HR = 2.11, 95% CI:1.23–3.64, *P* = 0.007), Table 3. Kaplan-Meier curves were used to present the relationship between the two events and diabetes for a different view (Log-rank test: *P* = 0.000, *P* = 0.026, respectively,), Figs. 3, 4.

### Treatment and management

Patients in diabetes group treated by taking diabetes medications. A total of 1113 patients included, standard OSA treatment (CPAP) has been excluded, and patients with other therapy of OSA, including surgery (*n* = 49), weight loss (*n* = 142) and oral appliance therapy (*n* = 54) were included in our study. We have compared the impact of these therapy methods on the primary outcome (MACE) in supplementary Table S-2. In both groups, our study founded that patients undergone oral appliance therapy for OSA fell short of statistical significance for the risk of MACE compared to patients of OSA without undergone oral appliance therapy, Whereas patients of OSA without undergone weight loss or surgical treatment had a higher risk of MACE than patients with OSA with weight loss or surgical treatment, Table S-2. Table S-3 presents the prevalence of type 2 diabetes was higher in OSA patients without underwent weight loss or surgical treatment than OSA patients with weight loss or surgical treatment.

## Discussion

In our cohort study, OSA participants with diabetes had a higher incidence of all end events during the median 42-month follow-up. After adjusting for a range of potential confounders, our study showed a trend of increased risk for MACE, hospitalisation for unstable angina and a composite of all events in OSA patients with type 2 diabetes. Subgroup analysis demonstrated that adjusted hazard ratios for MACE by diabetes were higher in obese and overweight females ≥70 years and patients with mild OSA.

OSA is the most common type of sleep apnoea. It is caused by intermittent upper airway obstruction during sleep, resulting in repeated oxygenated haemoglobin desaturation and sleep fragmentation [26]. Multiple mechanisms link OSA to CVD complications, including insulin resistance, oxidative stress, sympathetic activation, endothelial dysfunction and increased inflammation [27]. A previous study showed a strong correlation between OSA and cardiac metabolic syndrome. However, this was a retrospective study of young and middle-aged adults [28]. The RICCADSA study also confirmed that OSA was an independent risk factor for poor cardiovascular prognosis in patients with acute coronary syndrome [29]. A meta-analysis revealed that the risk of fatal or non-fatal cardiovascular events in OSA patients was 3 times higher than controls [30]. Another study concluded that the mortality of ST-segment elevation MI in OSA patients was lower than non-OSA patients as OSA could initiate the mechanism of ‘ischaemic preconditioning’ to protect the myocardium [15]. There is a growing amount of evidence that the evolution of OSA severity is related to a deterioration in blood glucose control [31, 32]. Therefore, our study further investigated the impact of concomitant type 2 diabetes on the long-term risk of MACE in patients with OSA. Notably, our study provided significant findings as a multicentre OSA population-based study adjusted for several potential confounders with confirmed statistical significance for MACE between patients with and without diabetes.

OSA could activate numerous endothelial cells and inflammatory cells and result in endothelial dysfunction, a predictor for MACE [33]. A cohort study proved that severe OSA was associated with cardiovascular events [34]. However, a cross-sectional study confirmed that moderate-severe OSA had no effect on microvascular endothelial function, especially in patients with type 2 diabetes [11]. Statistically speaking, although our data showed no correlation between the evolution of OSA severity and MACE risk in patients with type 2 diabetes, the risk trend for MACE increased in mild OSA patients with type 2 diabetes, which is partly consistent with previous study findings. First, age may be a significant interference factor in the results of this study. Second, severe OSA may involve more effective self-protective mechanisms, such as excessive respiratory effort and/or increased respiratory frequency, compensating for hypoxia in the body to reduce MACE risk.

OSA and all-cause mortality were significantly associated with each other in the general population. A study found that intermittent hypoxia could have protective effects on the cardiovascular system in elderly patients with OSA, reducing the risk of cardiovascular death and all-cause mortality [14]. Our findings showed that type 2 diabetes was nominally associated with the incidence of all-cause mortality and fell short of statistical significance, possibly because 87.6% of OSA patients with diabetes in our study were in stable condition with no target organ damage. Even so, the potential impact of the complications of diabetes on all-cause mortality and cardiovascular death in OSA patients cannot be ignored, especially in clinical diagnosis and treatment. Edwards et al. demonstrated that the severity of hypoxia caused by OSA in elderly patients is lower than in young patients [35]. Our data showed that the risk of MACE in elderly OSA ≥70 years with concomitant diabetes was significantly higher than in patients below 70 years, possibly due to the complex symptoms in elderly patients and impaired hypoxia tolerance. This study revealed that type 2 diabetes was associated with a higher risk of MACE in overweight and obese patients with OSA, which is not in line with previous studies. However, the ‘obesity paradox phenomenon’ indicated that obese patients with cardiovascular disease had a better cardiovascular prognosis than non-obese patients [36]. It is essential to regulate the body mass index, especially in elderly OSA patients with concomitant diabetes.

Evidence reveals that OSA and type 2 diabetes are independent risk factors for cardiovascular disease [1]. Previous studies showed that patients with OSA had a higher risk of cardiovascular disease [37, 38]. However, a prospective survey of an Asian population showed no correlation between OSA and cardiovascular disease [39]. Adderley and Subramanian suggested that prevalent diabetes or incident diabetes during the follow-up period showed a higher CVD risk in OSA patients. However, in this study, most subjects were young and middle-aged patients in the UK [1]. Our study found that the elderly OSA patients with diabetes had a higher risk of MACE, especially females. Therefore, the relationship between diabetes and cardiovascular disease in OSA patients is worthy of further research.

### Study limitations

Our study has several strengths and a few limitations. First, we assessed the risk of CVD and all-cause mortality in the diabetes group and the non-diabetes group of OSA patients without including healthy controls. Second, a median follow-up period of 42 months may be insufficient for all end events development in this cohort. Although this was a multicentre prospective cohort study, the study population consisted of Chinese patients; hence, selection bias could occur. However, these limitations do not affect the value of our study.

## Conclusion

In conclusion, OSA and type 2 diabetes are interrelated and synergistic with MACE, hospitalisation for unstable angina and a composite of all events development. In the subgroup analysis, overweight and obese females, 70 years of age, with mild OSA and concomitant diabetes presented a higher risk of MACE. Physicians need to recognise that patients with OSA complicated with type 2 diabetes constitute a high-risk population requiring strategy implementation to detect type 2 diabetes and prevent vascular complications. Further large-scale cohort studies examining the correlation between OSA, diabetes and cardiovascular disease risk are needed.

## Supplementary Information

**Additional file 1: Supplementary Table s**-**1.** Characterics of covariates. **Supplementary Table s**-**2.** Subgroup analysis of the associations between type 2 diabetes and MACE. **Supplementary Table s**-**3.** Crude number of type 2 diabetes in treatment for OSA

## Data Availability

Our data may not be shared directly, because it is our teamwork; informed consent should be attained from all the team members. Our data or material may be available after contacting the corresponding author or first author.

## Abbreviations

CVD: Cardiovascular disease
OSA: Obstructive sleep apnoea
MACE: Major adverse cardiovascular events
PSG: Polysomnography
AHI: The apnoea-hypopnoea index
BMI: Body mass index
SBP: Systolic blood pressure
DBP: Diastolic blood pressure
ODI: The oxygen desaturation index
MSpO2: The mean pulse oxygen saturation
LSpO2: The lowest pulse oxygen saturation
CHD: Coronary heart disease
COPD: Chronic obstructive pulmonary disease
MI: Myocardial infarction
CPAP: Continuous positive airway pressure
AHR: Adjusted hazard ratios
HR: Hazard ratios
CI: Confidence intervals
HTN: Hypertension
