# Supplementary material for: Impact of obstructive sleep apnea complicated with type 2 diabetes on long-term cardiovascular risks and all-cause mortality in elderly patients
Source: BMC Geriatr. 2021 Sep 25;21:508. doi: 10.1186/s12877-021-02461-x (PMC8466658; doi:10.1186/s12877-021-02461-x)
Supplement: Supplementary file 1 — Additional file 1: Supplementary Table s-1. Characterics of covariates. Supplementary Table s-2. Subgroup analysis of the associations between type 2 diabetes and MACE. Supplementary Table s-3. Crude number of type 2 diabetes in treatment for OSA [file 12877_2021_2461_MOESM1_ESM.docx]

Supplementary Material

Supplementary Table s-1. Characterics of covariates

| Covariates | Category | Definition |
| --- | --- | --- |
| Sex, n (%) | Categorical variables | Male, Female |
| Age, year | Continuous variable |  |
| BMI, kg/m^2^ | Continuous variable |  |
| SBP, mmHg | Continuous variable |  |
| DBP, mmHg | Continuous variable |  |
| Smoking, n (%) | Categorical variables | Never smoking, Ever or Current smoking |
| Drinking, n (%) | Categorical variables | Never alcohol drinking, ever or Eurrent alcohol drinking |
| Plasma glucose, mmol/L | Continuous variable |  |
| HbA1c, % | Continuous variable |  |
| HbA1, mmol/L | Continuous variable |  |
| Waist circumference, cm | Continuous variable |  |
| Neck circumference, cm | Continuous variable |  |
| Waist/hip ratio | Continuous variable |  |
| TST, h | Continuous variable |  |
| AHI, events/h | Continuous variable |  |
| ODI, events/h | Continuous variable |  |
| MSpO_2_, % | Continuous variables |  |
| LSpO_2_, % | Continuous variables |  |
| T90, % | Continuous variables |  |
| time of SO_2_ ≤90%, min | Continuous variable |  |
| maximum apnea time, s | Continuous variables |  |
| average apnea time, s | Continuous variable |  |
| Severity of OSA, n (%) | Categorical variables | Mild OSA，Moderate OSA and Severe OSA |
| CHD , n (%) | Categorical variables | Yes, No |
| Hyperlipidemia, n (%) | Categorical variables | Yes, No |
| Hypertension, n (%) | Categorical variables | Yes, No |
| Atrial fibrillation, n (%) | Categorical variables | Yes, No |
| Carotid atherosclerosis, n (%) | Categorical variables | Yes, No |
| COPD, n (%) | Categorical variables | Yes, No |

Supplementary Table S-2. Subgroup analysis of the associations between type 2 diabetes and MACE

|  | Unadjusted analysis | | Adjusted analysis | |
| --- | --- | --- | --- | --- |
|  | *HR* (95*%CI* ) | *P*-Value | *HR* (95*%CI* ) | *P*-Value |
| Weight loss （n=142) |  |  |  |  |
| yes | 2.71 (0.60-12.22) | 0.194 | 0.73 (0.076-6.68) | 0.767 |
| no | 1.830 (1.18-2.843) | 0.007 | 1.60 (1.03-2.49) | 0.037 |
| Oral appliance therapy（n=54) |  |  |  |  |
| yes | 1.74 (0.45-6.67) | 0.420 | 1.42 (0.31-6.46) | 0.649 |
| no | 1.58 (0.977-2.56) | 0.062 | 1.57 (0.97-1.51) | 0.067 |
| Surgical treatment （n=49) |  |  |  |  |
| yes | 2.65 (0.532, 12.34) | 0.241 | 2.50 (0.425, 14.72) | 0.311 |
| no | 1.68 (1.06, 2.66) | 0.028 | 1.64 (1.03, 2.61) | 0.036 |

*variables used for adjustment model related to baseline or follow up parameters of patients (sex, BMI, plasma glucose, alcohol use, HbA1c, ODI, TST, T90, TSA90, WHR, waist circumference, AHI, average apnea time, maximum apnea time, and comorbidities of CHD, hyperlipidemia, hypertension, carotid atherosclerosis, atrial fibrillation and diabetes).

Supplementary Table S-3 Crude number of type 2 diabetes in treatment for OSA

|  | Diabetes (n=266) | Non-diabetes (n=847) |
| --- | --- | --- |
| Weight loss （n=142) |  |  |
| yes | 33 (12.4) | 109 (12.9) |
| no | 233(87.60) | 738(87.1) |
| Oral appliance therapy（n=54) |  |  |
| yes | 39(14.7) | 15 (1.78) |
| no | 227(85.3) | 832(98.2) |
| Surgical treatment （n=49) |  |  |
| yes | 2 (0.75) | 47(5.5) |
| no | 264 (99.2) | 800 (94.5) |
